# Five facts about *Giardia lamblia*

**DOI:** 10.1371/journal.ppat.1007250

**Published:** 2018-09-27

**Authors:** Lenka Cernikova, Carmen Faso, Adrian B. Hehl

**Affiliations:** Laboratory of Molecular Parasitology, Institute of Parasitology, University of Zurich (ZH), Zurich, Switzerland; Washington University School of Medicine, UNITED STATES

## Fact 1: Infection with *Giardia lamblia* is one of the most common causes of waterborne nonbacterial and nonviral diarrheal disease

*G*. *lamblia* (syn. *intestinalis*, *duodenalis*) is a zoonotic enteroparasite. It proliferates in an extracellular and noninvasive fashion in the small intestine of vertebrate hosts, causing the diarrheal disease known as giardiasis. Virtually all mammals can be infected with *G*. *lamblia*, and epidemiological data point to giardiasis as a zoonosis [1]. Infections in humans may be asymptomatic or associated with diarrhea, malabsorption, bloating, abdominal pain, fatigue, and weight loss. Based on the latest figures provided by WHO, *G*. *lamblia* is the third most common agent of diarrheal disease worldwide with over 300 million reported cases per annum, preceded only by rotavirus and *Cryptosporidium parvum* and *hominis* in the most vulnerable target group of children under five years of age [2]. The prevalence of giardiasis in humans ranges from 2%–3% in industrialized countries, up to 30% in low-income and developing countries [3]. Giardiasis was formerly included in the WHO neglected diseases initiative and is directly associated with poverty and poor quality of drinking water [4]. Acute infection develops over a period of three weeks, peaking at eight days post infection. Generally, healthy hosts clear the infection within 2–3 weeks, whereas the occasional chronically infected host shows signs of villus and crypt atrophy, enterocyte apoptosis, and ultimately severe disruption of epithelial barrier function [5]. Infection with *G*. *lamblia* has also been linked to the development of irritable bowel syndrome and chronic fatigue [6].

## Fact 2: *G*. *lamblia* presents a simplified subcellular organization but is not a primitive eukaryote

To date, four types of endomembrane compartments have been identified in the *Giardia* trophozoite, namely: the endoplasmic reticulum (ER), the nuclei, terminally-differentiated mitochondrial remnants named mitosomes, and peripheral vacuoles (PVs) [7]. Encystation-specific vesicles (ESVs) constitute a fifth compartment present only in encysting cells. Extreme genomic divergence has led to frequent artefacts such as long-branch attraction in earlier phylogenetic studies [8]. Combined with observations of elements of prokaryotic metabolism and the absence of bona fide eukaryotic organelles such as the Golgi apparatus, endosomes, and mitochondria, this resulted in a misclassification of *G*. *lamblia* as a primitive eukaryote and a concomitant misinterpretation of its evolutionary history [9]. However, molecular paleontology approaches aimed at identifying machinery present in the last eukaryotic common ancestor (LECA) indicated that this organism likely possessed all extant eukaryotic organelles and corresponding trafficking pathways. This supports the notion that species with a simplified cellular organization such as the ancestor of *G*. *lamblia* evolved via a reduction of complexity, likely linked to adoption of a parasitic lifestyle, and that *G*. *lamblia* is therefore most likely not a primary primitive eukaryote but secondarily reduced. Interestingly, recent efforts aimed at rooting the eukaryotic tree place this root between the Excavata supergroup, to which *G*. *lamblia* belongs, and all other eukaryotes [10].

## Fact 3: *G*. *lamblia* feeds using specialized organelles called PVs

**Fig 1:**
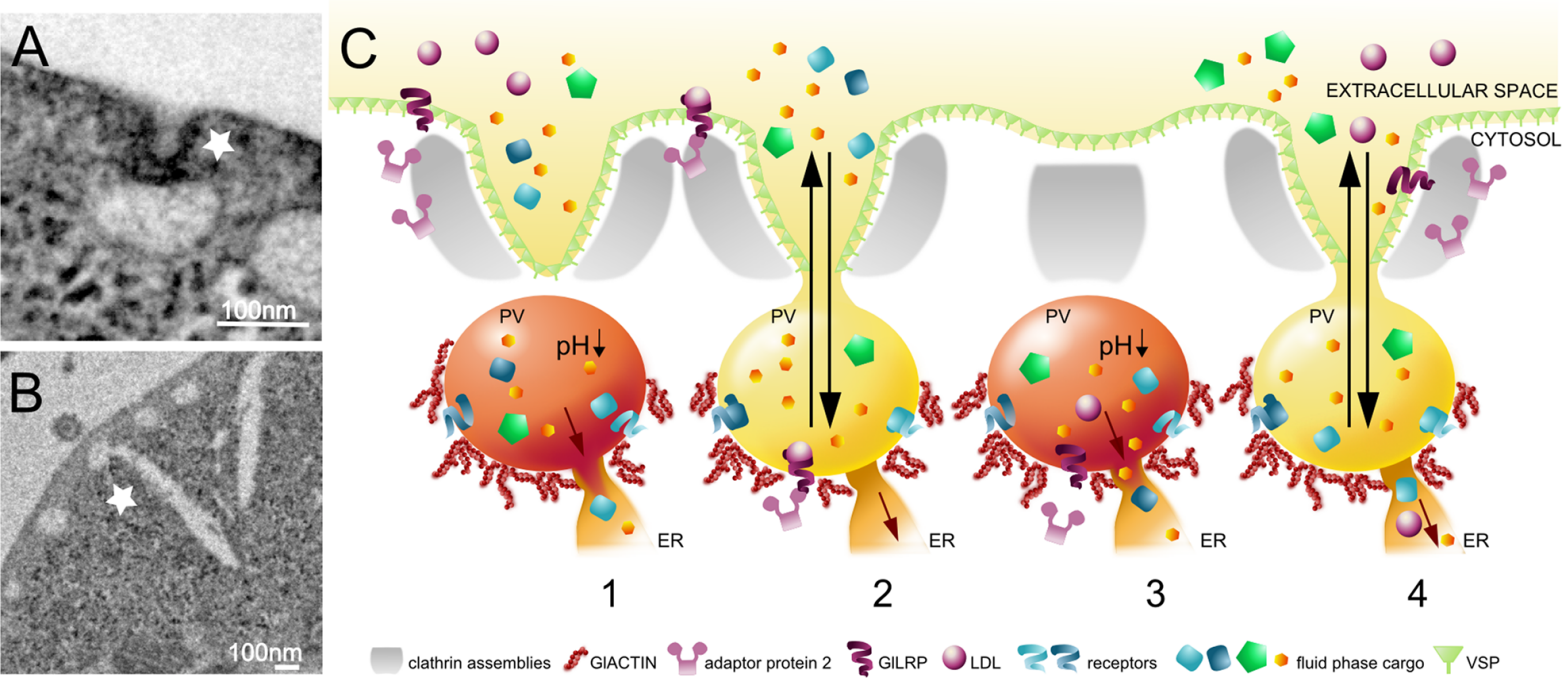
A working model for fluid-phase and receptor-mediated nutrient uptake through the PVs of *G*. *lamblia*. (A) A TEM image of a PV making contact with a PM-derived invagination (white star). (B) Connections between PVs and ER membranes (white star) are frequently detected in TEM tomograms. (C) The “kiss and flush” working model for fluid-phase and receptor-mediated endocytosis in PVs is based on previously published data [12,13,14,27] and is represented as a continuum of nutrient entry, retention, release, and transfer. (1) Acidifying PVs contain fluid-phase cargo, which is either free or retained by PV-resident receptors (blue ribbons). Released cargo travels further to the lumen of connecting ER tubules. Some cargoes (green pentagon) can be excluded from further passage to the ER. (2) Uptake is mediated by fusion between the VSP-coated PM and the PV membrane through PM-derived invaginations surrounded by clathrin arrays associated to AP2 complexes. This event corresponds to the “kiss” phase in which formation of a channel allows exchange of fluid-phase material between the PV lumen and the extracellular space. Usable nutrients may move freely or be bound by receptors lining the organelle lumen, whereas useless or harmful molecules may be released back in the extracellular space during the “flush” phase. LDL receptor (GILRP) traffics in an AP2-dependent manner from the PM (1) to the PV membrane. (3) PV–PM luminal continuity terminates and a new round of receptor-bound nutrient release mediated by intralumenal acidification ends with further passage to connected ER. (4) The PV is now ready for another round of endocytosis and exchange with the extracellular environment and GILRP is recycled back to the cell’s surface. ER, endoplasmic reticulum; GILRP, giardial putative low-density lipoprotein receptor; LDL, low-density lipoprotein; PM, plasma membrane; PV, peripheral vacuole; TEM, transmission electron microscopy; VSP, variant surface protein.

Due to streamlining of most anabolic pathways, *G*. *lamblia* cells are highly dependent on nutrient uptake from the host’s gut by means of an array of organelles, i.e., PVs (Fig 1A). This active host–pathogen interface is at the crossroads of both endo- and exocytic trafficking in *G*. *lamblia* trophozoites (Fig 1B). The main function of PVs is to periodically endocytose fluid-phase extracellular material and to expel harmful or unusable substances into the environment again. This is in contrast to the unidirectional endocytic uptake of fluid-phase material via cytostome-like structures in many protozoa as well as in *Spironucleus* spp., the closest known relatives of *Giardia* [11]. PVs effectively act as “safety-lock" compartments for efficient environmental sampling, i.e., the uptake and intracellular sorting of gut content. The “kiss and flush” working model (Fig 1C) developed in our group is based on experimental data suggesting that PV membranes and the plasma membrane (PM) transiently fuse (the “kiss” phase) and become continuous, thereby generating an opening to the extracellular space allowing exchange of fluid-phase material between the PV lumen and the environment [12]. This PV–PM connection is then resolved, and sorting of usable nutrients occurs within the acidifying PV lumen. Any material that is not retained would then be released back into the extracellular space in a new round of PV–PM connection (the “flush” phase). The flushing of the PV lumen makes exchange of fluid-phase material bidirectional and likely compensates for the lack of bona fide lysosomes as endpoints of endocytic transport. Endocytosis through PVs is likely the main route of nutrient uptake into the *Giardia* cell, although there are isolated reports on receptor-mediated uptake of lipid particles. The giardial putative low-density lipoprotein (LDL) receptor (GILRP; purple ribbon in Fig 1C) in *G*. *lamblia* was shown to interact with AP2 components [13], although its exact trafficking mechanism remains uncharacterized (Fig 1C). Short actin filaments were also shown to be involved in LDL uptake and were localized in close proximity to PVs [14].

## Fact 4: *G*. *lamblia* survives in the environment as infectious cysts

Completion of the life cycle by transmission of *G*. *lamblia* to a new host requires no vectors and is based on the alternation of a vegetative stage, the trophozoite, and an environmentally resistant infectious stage—the cyst. The cyst is the only stage of *G*. *lamblia* able to survive outside of the host and is responsible for the initiation of a new infectious cycle. Cyst development may already begin in the small intestine of parasitized hosts when a variable fraction of proliferating trophozoites initiates a cellular differentiation program called encystation [15]. Laboratory protocols for inducing encystation include lipid depletion and an increase in culturing medium pH over a period of ca. 20–24 hours [16]. The assumption is that these conditions mimic decreasing lipid availability and ascending pH gradients naturally present along the gastrointestinal tract. During encystation, flagellated pear-shaped binucleated trophozoites undergo dramatic morphological and biochemical cellular remodeling, culminating in the formation of nonflagellated oval quadrinucleated cysts, surrounded by a cyst wall (CW). The CW is composed of a thick mesh of cyst wall proteins (CWPs) complexed to a unique sugar polymer of β1,3-linked-N-acetylgalactosamine [17] and virtually shields the cyst’s interior from any solvent. Deposition of the CW is a tightly-regulated event that occurs exclusively in encysting trophozoites and requires neogenesis of specialized secretory organelles called ESVs [18]. ESVs traffic, sort, and modify mainly CWPs from their initial site of deposition at the ER en route to the parasite cell’s surface. Elimination of a single CWP by complete gene disruption was shown to abolish CW formation altogether [19]. The exact in vivo stimuli for this process are not yet well known, although recent studies on the dynamics of encystation in animal models point towards a link between high-density focal trophozoite populations in the proximal small intestine and encystation [15]. These findings argue against the natural pH and lipid gradients being the sole external triggers for differentiation. In turn, this raises the interesting possibility that trophozoites can sufficiently alter the local environment to generate conditions favorable for triggering differentiation.

Cysts are then shed through host feces and were reported to remain viable for several months in water at temperatures below 10 °C and several weeks at room temperature [20]. Based on experimental gerbil (*Meriones ungulatus*) infections, the minimal infectious dose is less than 10 cysts [21]. Once viable cysts are ingested, passage through the stomach and physical stimuli (temperature, pH) initiate a cellular program termed excystation in which both host and parasite proteases collaborate to degrade the CW, allowing the short-lived excyzoite to escape and rapidly divide twice to give rise to four trophozoites [22]. In vitro (and most likely in vivo) this process is completed within minutes [23].

## Fact 5: Novel rational design-based vaccination strategies against *G*. *lamblia* are yielding encouraging results

Currently, treatment of giardiasis in humans is based almost exclusively on administration of antiprotozoals belonging to the family of 5-nitroimidazoles, whereas infected animals are treated with benzimidazoles. A crude veterinary vaccine called GiardiaVax for nonchronically infected dogs and cats was already licensed [24]. However, it does not inhibit trophozoite proliferation and in many cases has to be combined with additional drug treatment. In recent years, work done especially by the Lujan group at the Universidad Católica de Córdoba in Argentina has uncovered the potential for variant surface proteins (VSPs) as effective vaccination antigens in companion animals. The entire trophozoite surface is covered by a dense coat of VSP anchored in the plasma membrane with a C-terminal hydrophobic transmembrane anchor sequence. From a repertoire of over 200 homologous genes encoded in the parasite genome, only one VSP is expressed on the surface of every single trophozoite at any given moment. Antigenic switching and regulation of VSP expression were shown to occur by RNAi-like mechanisms that could be perturbed to deregulate VSP production, leading to trophozoites exposing the full VSP panel on their surface [25]. Oral vaccination trials on gerbils showed that animals initially infected with deregulated cells expressing all of the VSPs encoded in their genome are largely protected from challenge infections by *Giardia* clones that express a unique VSP on their surface or by cysts obtained from infected individuals [25]. The same vaccine was tested on cats and dogs, showing high efficiency in preventing new infections and reducing chronic giardiasis in domestic animals both in experimental and natural infections [26]. These data are based on the rational design of vaccination strategies underpinned by a deep understanding of *G*. *lamblia*’s molecular and cell biology and hold great promise for eradication of giardiasis in both animals and humans.
